# Effects of Dietary Cooked Navy Bean on the Fecal Microbiome of Healthy Companion Dogs

**DOI:** 10.1371/journal.pone.0074998

**Published:** 2013-09-11

**Authors:** Katherine R. Kerr, Genevieve Forster, Scot E. Dowd, Elizabeth P. Ryan, Kelly S. Swanson

**Affiliations:** 1 Division of Nutritional Sciences and Department of Animal Sciences, University of Illinois, Urbana, Illinois, United States of America; 2 Department of Environmental and Radiological Health Sciences, Colorado State University, Fort Collins, Colorado, United States of America; 3 MR DNA (Molecular Research LP), Shallowater, Texas, United States of America; Naval Research Laboratory, United States of America

## Abstract

**Background:**

Cooked bean powders are a promising novel protein and fiber source for dogs, which have demonstrated potential to alter microbial composition and function for chronic disease control and prevention. This study aimed to determine the impact of cooked navy bean powder fed as a staple food ingredient on the fecal microbiome of healthy adult pet dogs.

**Methodology/Principal Findings:**

Fecal samples from healthy dogs prior to dietary control and after 4 wk of dietary treatment with macro- and micronutrient matched diets containing either 0 or 25% cooked navy beans (n = 11 and n = 10, respectively) were analyzed by 454-pyrosequencing of the 16S rRNA gene. There were few differences between dogs fed the control and navy bean diets after 4 wk of treatment. These data indicate that there were no major effects of navy bean inclusion on microbial populations. However, significant differences due to dietary intervention onto both research diets were observed (i.e., microbial populations at baseline versus 4 wk of intervention with 0 or 25% navy bean diets). After 4 wk of dietary intervention on either control or navy bean diet, the Phylum Firmicutes was increased and the Phyla Actinobacteria and Fusobacteria were decreased compared to baseline.

**Conclusions:**

No negative alterations of microbial populations occurred following cooked navy bean intake in dogs, indicating that bean powders may be a viable protein and fiber source for commercial pet foods. The highly variable microbial populations observed in these healthy adult pet dogs at baseline is one potential reason for the difficulty to detect alterations in microbial populations following dietary changes. Given the potential physiological benefits of bean intake in humans and dogs, further evaluation of the impacts of cooked navy bean intake on fecal microbial populations with higher power or more sensitive methods are warranted.

## Introduction

A more in depth understanding of the companion canine microbiome, including the phylogenetic structure, functional capacity, and how they are altered by diet, is needed. Evidence supports a role of the gut microbiota in digestion and energy harvest, defense against pathogens, nutritional support for enterocytes, and immune stimulation. Molecular approaches, including 454-pyrosequencing, have shown utility for improving our understanding of the complex and diverse microbial communities that exist in the gut. Results from studies characterizing the canine fecal microbiota were recently reviewed [1]. Hundreds of species are present, but most are members of the predominant phyla Firmicutes (27 to 97% of sequences), Fusobacteria (< 1 to 50% of sequences), and Bacteroidetes (< 1 to 19% of sequences). 

Few studies have examined the impact of diet on canine fecal microbial populations utilizing 454-pyrosequencing and metagenomic techniques [2], [3], [4], [5]. Most of these studies have focused on alterations by dietary fibers, prebiotics, and synbiotics. To our knowledge, dry beans have not been included in any commercial canine pet foods, and are therefore a novel protein and fiber source for canines. In addition, dry beans are an excellent source of minerals, essential vitamins, and bioactive compounds [6]. We recently reported the safety and digestibility of cooked navy bean powders from a placebo-controlled intervention trial in client-owned dogs [7]. Additionally, we have demonstrated that cooked bean intake regulated lipid metabolism during weight loss in overweight and obese dogs [8]. However, it is unknown how navy beans, when used as a major source of protein and fiber in the diet (> 25%), may alter the canine gut microbiota when compared to commercial fiber sources.

Given that the composition, dynamics, and functionality of the canine gut microbiota have been largely evaluated in colonies of research dogs living in controlled environments, we sought to examine the influence of a bean-based diet on the gut microbiome of healthy free-living client-owned dogs. Control and navy bean-based diets were formulated and matched for total macronutrient and micronutrient content. This approach allowed us to test whether the source of these nutrients and the phytochemicals present in navy beans were responsible for changes in the fecal microbial communities.

## Materials and Methods

### 2.1 Animals

The Colorado State University (CSU) Institutional Animal Care and Use Committee approved all trial operations, animal care procedures, and collection of biological samples for safety and digestibility of experimental research diets before beginning study (Protocol ID # 10-1932A).

Twenty-one clinically healthy, adult, normal weight, male and female client-owned dogs were recruited to participate in a randomized, double-blinded, and placebo-controlled canine dietary intervention study at the CSU Veterinary Teaching Hospital’s Animal Cancer Center as described by [7]. Briefly, male and female dogs of any breed between the ages of 2 and 7 yr that had body condition scores (BCS) between 4 and 7 on a 9-point scale (determined by study clinician [9]), and weighed at least 10 kg were eligible to participate. Body condition score is a semiquantitative assessment of body composition based on palpation and visual assessment that has been demonstrated to correlate with dual-energy X-ray absorptiometry (DEXA) evaluations of body fat content in dogs [10]. For this study, the 9 point scale was used where dogs with a BCS of 4 and 5 are considered ideal weight, 6 and 7 are overweight, and 8 and 9 are obese [9]. Dogs were excluded if they had any reported dietary allergies, intestinal sensitivities/discomforts, or prior history of cancer. Dogs must not have taken antibiotics or analgesics for at least 1 mo before starting the study. Preventive medications (e.g., heartworm prevention) were allowed. Dog owners were screened and provided informed consent before their pet’s enrollment at the CSU Animal Cancer Center. Animal data are presented in Table 1.

**Table 1:** Information about the dogs that participated in the study.

Number	Diet^1^	Breed	Sex	Age (yr)	BCS^2^
1	CON	Australian Cattle Dog	F	2	4
2	CON	Australian Cattle Dog	M	2	5
3	CON	Golden Retriever	F	5	5
4	CON	Hound Mix	M	2	5
5	CON	Lab	M	3	5
6	CON	Mixed	F	5	7
7	CON	Mixed	M	3	5
8	CON	Pitbull Mix	F	3	5
9	CON	Pitbull Mix	M	4	5
10	CON	Retriever	M	2	6
11	CON	St. Bernard	F	4	5
12	NB	Australian Cattle Dog	F	2	6
13	NB	Australian Cattle Dog	M	2	5
14	NB	Dalmatian	F	6	5
15	NB	Golden Retriever	M	4	5
16	NB	Mixed	F	3	4
17	NB	Mixed	F	5	5
18	NB	Pointer	M	5	4
19	NB	Shep/Airelale x	M	7	7
20	NB	Standard Poodle	F	3	5
21	NB	Terrier	F	4	5

1 CON  =  diets containing 0% cooked navy bean powder (n = 11); NB  =  diets containing 25% cooked navy bean powder (Vegeful ADM Edible Bean Specialties, Decatur, IL) (n = 10).

2 BCS  =  body condition score.

### 2.2 Dietary Treatments

In depth diet description is available in [7]. Dogs were fed one of two dietary treatments: 1) control diet [CON (ADM Alliance Nutrition, Quincy, IL)]; or 2) navy bean diet [NB (ADM Alliance Nutrition)]. The NB included 25% cooked navy bean powder (Vegeful ADM Edible Bean Specialties, Decatur, IL). Adjustments of other ingredients (e.g., wheat and corn) in the NB were made to match the macronutrient, micronutrient, and total caloric content of the CON (crude protein: 30% DM; fat: 14% DM). Diets were formulated to meet the nutrient requirements of domestic dogs [11].

Dogs were fed to maintain a stable body weight (less than 2% change per wk). Owners were instructed to feed only the research diet in the prescribed daily amount. Food offered and refused was measured and recorded by owners daily. Any intake besides that instructed was included in the owner records. Water was provided ad libitum.

### 2.3 Sample Collection and Analysis

Fecal samples were collected at baseline and after 4 wk of treatment. Fecal samples were collected within 5 h of being voided and were stored at −20°C until shipment for analysis.Genomic DNA was extracted using the repeated bead beater method described by Yu and Morrision [12] with a DNA extraction kit (QIAamp DNA Stool Mini Kit, Qiagen, Valencia, CA). Once the DNA was eluted, all samples were purified using the QIAquick PCR Purification Kit (Qiagen) according to the manufacturer’s instructions. Extracted DNA was quantified using a spectrophotometer (NanoDrop ND-1000, Nano-Drop Technologies, Wilmington, DE). Genomic DNA was diluted to 20 ng/µl and amplification of a 600-bp region of the V4−V6 variable region of the 16S rRNA gene was done using barcoded primers according to Cephas et al. [13]. The barcoded primers contained a forward ‘LinkerA’ sequence (CGTATCGCCTCCCTCGCGCCATCAG), a reverse ‘LinkerB’ sequence (CTATGCGCCTTGCCAGCCCGCTCAG), a multiplex identifier (MID) unique to each sample, and a Eubacterial-specific sequence [530F (5′-GTGCCAGCMGCNGCGG) or 1100R (5′-GGGTTNGNTCGTTG)] for the V4−V6 region of the 16S rRNA gene. PCR reactions of 25 µl were performed for each sample using a barcoded forward primer (10 µM), barcoded reverse primer (10 µM), dNTP mix (10 mM), FastStart 10X buffer with MgCl2, fastStart HIFi Polymerase (5U/µl) and 5 µl of genomic DNA. The PCR conditions were: 94°C for 3 min; 32 cycles of 94°C for 15s, 62°C for 45s, and 72°C for 1 min extension; followed by 72°C for 8 min. PCR amplicons of all samples were further purified using AMPure XP beads (Beckman-Coulter, Inc., Brea, CA) to remove smaller fragments. Finally, the amplicons were combined in equimolar ratios to create a DNA pool that was used for pyrosequencing. Pyrosequencing of the PCR amplicons was performed at the W. M. Keck Center for Biotechnology at the University of Illinois using a 454 Genome Sequencer and FLX titanium reagents (Roche Applied Science, Indianapolis, IN). After sequencing was completed, all reads were scored for quality and any poor quality reads and primer dimers were removed.

### 2.4 Data Analysis

The sequences were selected to estimate total bacterial diversity of DNA samples in a comparable manner and were trimmed to remove barcodes, primers, chimeras, plastid, mitochondrial, any non-16S bacterial reads, and sequences < 350bp. Sequences were then denoised and chimeras removed using Black Box Chimera Check (B2C2) [14]. A total of 4500 +/− 100 rarified sequences from each sample were selected based upon highest average quality score, sequences trimmed to 350bp, and aligned with MUSCLE [15] and then distance matrix was calculated from the alignment with PHYLIP (PHYLogeny Inference Package). Operational Taxonomical Units (OTU) were defined as clustering at 3% divergence (97% similarity) using the read.otu command by MOTHUR [16].

Bacterial ID community structure was evaluated using Phred25 quality reads, including both 530F and 1100R oriented (each analyzed separately), trimmed to remove tags and primer sequence collections, then depleted of chimera, plastid, mitochondrial, sequences with ambiguous base calls, sequences with >6bp homopolymer runs, and any non-16S reads (<70% identity to any known high quality 16S sequence) and sequences < 250bp. The final sequence data were evaluated using Kraken (www.krakenblast.com) against a 06-12-11 version database curated from NCBI to include >350,000 high quality 16S bacteria and archaeal sequences as well as quality control screening sequences for mitochondria, plastid, and chloroplast screening sequences. Blast output based upon top hit designations were compiled to generate percentage files at each taxonomic level as described previously [14], [17]. 

UniFrac was utilized to evaluate the relatedness of samples [13]. The high quality 1100R sequences prepared as described above were aligned utilizing MUSCLE, and an optimized tree was generated. This tree served as the input tree for UniFrac. Weighed and normalized Principal Component Analysis (PCA) was performed to evaluate similarity among samples, where each sample represents an environment. Primary 3 vectors significance was evaluated using two tailed T-test.

Pyrosequencing data are presented as percentage of sequences at each taxonomic level and were analyzed using the MIXED procedure of SAS (version 9.2, SAS Institute Inc., Cary, NC). For 4-wk microbial data, the fixed effect of treatment was tested. Means were separated using LSD with a Tukey adjustment to control for multiple comparisons. Post hoc contrasts were utilized to compare 0-wk vs. 4-wk microbial data. A probability of P≤0.05 was accepted as being statistically significant and P≤0.10 was accepted as a trend.

## Results

Pyrosequencing of 16S rRNA barcoded amplicons resulted in a total of 560,308 high-quality sequences, with an average of 13,340 sequences per sample. Sequences are available at the NCBI sequence read archive (http://www.ncbi.nlm.nih.gov/Traces/sra/) under accession numbers SAMN02117000 to SAMN02117041. The PCA revealed no separation due to treatment at any taxonomic level (data not shown). The OTU, ACE, and Chao estimates of diversity were not different (P>0.05) among groups (data not shown). Similarly, diet did not have a significant impact on the Shannon Index, which indicates that the consumption of navy bean did not alter the overall fecal bacterial diversity at 4 wk.

### 3.1 Predominant Taxa in Client-Owned Dogs

Fourteen phyla were identified in the feces. The phyla Actinobacteria, Bacteroidetes, Firmicutes, Fusobacteria, and Proteobacteria were present in a majority of dogs at both time points (80 to 100% of dogs). The remaining phyla (Chloroflexi, Cyanobacteria, Deferribacteres, Deinococcus Thermus, Gemmatimonadetes, Spirochaetes, Tenericutes, Spirochaetes, Verrucomicrobia, and TM7) were present in only a few (1 to 3) dogs and were present as < 0.1% of all bacterial sequences. Firmicutes was the most abundant bacterial phylum (60 to 95% of all bacterial sequences; mean ± SD: 82.9±9.6%). Fusobacteria and Actinobacteria were the next most abundant phyla (0 to 28% of all bacterial sequences; mean ± SD: 7.5±8.0%; and 1 to 30% of all bacterial sequences; mean ± SD: 7.66±6.8%, respectively). Proteobacteria and Bacteroidetes contributed to about 0 to 13% and 0.01 to 5.8% of all bacterial sequences, respectively.

Firmicutes was largely comprised of the classes Clostridia (33 to 86% of all bacterial sequences; mean ± SD: 64.8±11.2%), Bacilli (0 to 45% of all bacterial sequences; mean ± SD: 3.8±9.3%), and Erysipelotrichi (4 to 38% of all bacterial sequences; mean ± SD: 15.3 ±8.1%).

The Bacilli class was predominantly comprised of the genera *Lactobacillus* (0 to 42% of all bacterial sequences; mean ± SD: 1.6±7.0%), *Streptococcus* (0 to 29% of all bacterial sequences; mean ± SD: 1.2±4.7%) and *Enterococcus* (0 to 26% of all bacterial sequences; mean ± SD: 0.85±4.0%). Within the class Clostridia, the major genera were: *Clostridium* (11 to 48% of all bacterial sequences; mean ± SD: 26.4±8.8%), *Blautia* (4 to 30% of all bacterial sequences; mean ± SD: 16.5±6.6%), *Ruminococcus* (3 to 27% of all bacterial sequences; mean ± SD: 11.0±4.5%), and *Faecalibacterium* (0 to 17% of all bacterial sequences; mean ± SD: 3.5±4.6%). Within the class Erysipelotrichi the major genera were *Catenbacterium* (0 to 7% of all bacterial sequences; mean ± SD: 4.8±7.0%) and *Allobaculum* (0 to 10% of all bacterial sequences; mean ± SD: 3.8±2.9%). Many species were present at an average of greater than 2% of all bacterial sequences and with a maximum greater than 10%: *Blautia glucerasea, B. hansenii, B. product*, *Clostridium hiranonis, Catenibacterium mitsuokai, Eubacterium biforme, Ruminococcus gnavus,* and *Megamonas funiformis*. However, none were present in all dogs at high proportions [e.g., *Clostridium hiranonis* accounted for 0.2 to 41% of all bacterial sequences (mean ± SD: 16.3±8.3%)].

The predominant genus in the Fusobacteria phylum was *Fusobacterium* (0 to 28% of all bacterial sequences; mean ± SD: 7.5±8.0%). The predominant genus from the Actinobacteria phylum was *Collinsella* (0.7 to 30% of all bacterial sequences; mean ± SD: 7.1±6.6%), and was primarily comprised of *C. intestinalis* (0.6 to 29% of all bacterial sequences; mean ± SD: 6.7±6.5%). The predominant genus in the Proteobacteria phylum was *Shigella* (0 to 10% of all bacterial sequences; mean ± SD: 0.5±1.7%). The predominant species was *Shigella sonnei* (0 to 5% of all bacterial sequences; mean ± SD: 0.3±1.0%).

### 3.2 Effects of Bean-Based Diet

There were no diet-induced differences detected at the phylum or family level (Table 2). The abundance of *Clostridium saccharogumia* was lower (P = 0.03) and *Clostridium ramosum* tended to be higher (P = 0.09) in dogs fed NB compared to those CON. Fecal *Coprobacillus catenformis* were lower (P = 0.03), while *Blautia wexlerae* was higher (P = 0.03) in dogs fed NB compared to those fed CON.

**Table 2:** Predominant bacterial phyla, family, genera, and species (expressed as percentage of sequences) in feces of dogs fed CON or NB.^1^

Phylum	Family	Genus	Species	CON	NB	Pooled SEM	P - Value
Actinobacteria				6.08	5.53	2.07	0.85
	Coriobacteriaceae			6.05	4.80	2.05	0.66
		Collinsella		5.75	4.59	1.99	0.68
			*Collinsella intestinalis*	5.39	4.26	1.90	0.68
	Bifidobacteriaceae			0.02	0.72	0.33	0.15
		Bifidobacterium		0.02	0.72	0.34	0.15
Bacteroidetes				0.78	0.74	0.35	0.93
	Bacteroidaceae			0.22	0.27	0.10	0.71
	Prevotellaceae			0.56	0.47	0.32	0.84
		Prevotella		0.56	0.47	0.32	0.84
			*Prevotella copri*	0.43	0.31	0.24	0.71
Firmicutes				87.53	86.81	2.71	0.85
	Clostridiaceae			28.42	23.32	2.71	0.19
		Clostridium		27.95	22.77	2.63	0.18
			*Clostridium citroniae*	0.17	0.28	0.10	0.48
			*C. colinum*	0.22	0.20	0.09	0.91
			*C. glycyrrhizinilyticum*	0.25	0.24	0.07	0.92
			*C. hathewayi*	0.31	0.56	0.20	0.37
			*C. hiranonis*	17.20	15.67	2.63	0.69
			*C. leptum*	0.43	0.42	0.23	0.98
			*C. nexile*	0.40	0.08	0.20	0.24
			*C. ramosum*	0.07	0.20	0.05	0.09
			*C. saccharogumia*	2.62	1.02	0.51	0.03
			*C. spiroforme*	1.39	0.89	0.31	0.27
	Unclassified Clostridiaceae			17.56	19.01	2.00	0.61
		Blautia		17.51	19.00	1.99	0.60
			*Blautia coccoides*	0.76	0.76	0.09	0.94
			*B. glucerasea*	4.76	4.52	0.83	0.83
			*B. hansenii*	4.35	5.76	1.31	0.45
			*B. producta*	5.36	4.31	0.64	0.25
			*B. schinkii*	1.67	0.97	0.56	0.78
			*B. wexlerae*	0.23	2.33	0.68	0.03
		Faecalibacterium		4.48	2.17	1.43	0.26
	Enterococcaceae			0.57	2.75	1.23	0.21
		Enterococcus		0.55	2.75	1.23	0.21
			*Enterococcus durans*	0.37	0.11	0.16	0.25
	Erysipelotrichaceae			19.27	15.67	2.45	0.30
		Allobaculum		4.75	3.26	0.89	0.25
			*Allobaculum stercoricanis*	4.75	3.26	0.89	0.25
		Catenibacterium		4.14	7.68	2.17	0.26
			*Catenibacterium mitsuokai*	4.02	7.53	2.13	0.25
		Coprobacillus		0.84	0.20	0.20	0.03
			*Coprobacillus cateniformis*	0.83	0.18	0.20	0.03
		Eubacterium		3.17	6.49	1.80	0.20
			*Eubacterium biforme*	2.87	6.26	1.82	0.19
			*E. dolichum*	0.29	0.22	0.11	0.66
	Eubacteriaceae			0.40	0.32	0.10	0.58
		Eubacterium		0.39	0.32	0.10	0.57
	Lachnospiraceae			0.61	0.35	0.15	0.24
		Lactonifactor		0.22	0.26	0.09	0.77
			*Lactonifactor longoviformis*	0.22	0.26	0.09	0.77
	Lactobacillaceae			0.14	4.56	2.16	0.15
		Lactobacillus		0.05	4.48	2.18	0.15
			*Lactobacillus gasseri*	< 0.01	0.44	0.20	0.14
			*L. reuteri*	< 0.01	0.39	0.19	0.14
			*L. rogosae*	0.04	0.23	0.15	0.37
	Peptococcaeae			0.50	0.43	0.19	0.82
		Peptococcus		0.42	0.38	0.20	0.90
			*Peptococcus niger*	0.42	0.38	0.19	0.90
	Ruminococcaceae			16.36	12.84	1.57	0.12
		Ruminococcus		11.53	10.42	1.43	0.59
			*Ruminococcus gnavus*	3.24	3.35	0.84	0.93
			*R. obeum*	0.31	0.29	0.05	0.78
	Streptococcaceae			3.05	0.50	1.45	0.22
		Streptococcus		3.04	0.41	1.43	0.20
			*Streptococcus equinus*	0.62	0.13	0.30	0.25
	Turicibacteriaceae			1.03	0.54	0.37	0.35
		Turicibacter		1.03	0.53	0.37	0.35
			*Turicibacter sanguinis*	0.70	0.36	0.28	0.40
	Veillonellaceae			4.45	3.68	1.65	0.83
		Dialister		1.76	< 0.01	1.00	0.22
		Megamonas		1.92	2.46	1.47	0.80
			*Megamonas funiformis*	1.89	2.40	1.43	0.80
		Megasphaera		0.27	0.38	0.57	0.23
			*Megasphaera elsdenii*	0.26	0.37	0.56	0.89
Fusobacteria				4.79	5.24	2.45	0.89
	Fusobacteriaceae			4.78	5.24	2.40	0.89
		Fusobacterium		4.78	5.24	2.40	0.89
			*Fusobacterium perfoetens*	0.21	0.17	0.17	0.86
			*F. ulcerans*	0.16	0.20	0.07	0.69
Proteobacteria				0.79	1.68	0.72	0.39
	Enterobacteriaceae			0.15	1.22	0.70	0.29
		Shigella		0.13	0.80	0.53	0.38
			*Shigella sonnei*	0.03	0.58	0.31	0.21

1 Data are presented as percentage of sequences. CON  =  diets containing 0% cooked navy bean powder (n = 11); NB  =  diets containing 25% cooked navy bean powder (Vegeful ADM Edible Bean Specialties, Decatur, IL) (n = 10).

### 3.3 Baseline vs. Treatment

There were significant changes in the fecal microbiota between baseline and wk 4 of dietary intervention regardless of diet fed (Table 3). At the phylum level, the proportion of Firmicutes was higher (P < 0.01), and the proportions of Fusobacteria and Actinobacteria were lower (P≤0.05) at 4 wk compared to baseline (Table 3). Within the phylum Firmicutes, the proportion of Lachnospiraceae tended to be lower (P = 0.09), while Erysipelotrichaceae tended to be higher (P = 0.09) at 4 wk compared to baseline. Within the family Lachnospiraceae, the proportions of Blautia and *Blautia coccoides* were higher (P≤0.01), the proportion of *B. producta* tended to be higher (P = 0.10) at 4 wk compared to baseline. Additionally, within the family Clostridiaceae, the proportion of *Clostridium leptum* was lower (P = 0.02), and the proportion of *C. indolis* tended to be lower (P = 0.06) at 4 wk compared to baseline. Within the Ruminococcaceae family, *Ruminococcus gnavus* was lower (P<0.01), while the proportion of *R. obeum* tended to be higher (P = 0.06) at 4 wk compared to baseline. Within the phylum Fusobacteria, the proportion of Fusobacteriaceae was lower (P≤0.04), and proportions of Fusobacterium and *Fusobacterium perfoetens* tended to be lower (P≤0.10) at 4 wk compared to baseline. Within the phylum Actinobacteria, the proportion of Coriobacteriaceae was lower (P = 0.05) and the proportions of Collinsella and *Collinsella intestinalis* tended to be lower (P = 0.06) at 4 wk compared to baseline.

**Table 3:** Select bacterial phylum, family, genus and species in the feces of dogs fed macro- and micronutrient matched dietary treatments for 4 wk.^1^

Phylum	Family	Genus	Species	Wk 0	Wk 4	Pooled SEM	P - Value
**Actinobacteria**				9.59	5.80	1.46	0.05
	Coriobacteriaceae			9.56	5.43	1.45	0.05
		Collinsella		8.94	5.17	1.40	0.06
			*Collinsella intestinalis*	8.57	4.82	1.38	0.06
**Firmicutes**				78.69	87.17	1.92	< 0.01
		Blautia		14.93	18.25	1.42	0.10
			*Blautia coccoides*	0.48	0.76	0.06	< 0.01
			*B. producta*	3.81	4.84	0.45	0.10
	Clostridiaceae			28.03	25.87	1.92	0.43
		Clostridium		27.40	25.36	1.90	0.45
			*Clostridium indolis*	0.09	0.01	0.03	0.06
			*C. leptum*	0.96	0.43	0.16	0.02
	Erysipelotrichaceae			13.21	17.47	1.74	0.09
	Lachnospiraceae			0.74	0.48	0.11	0.09
	Ruminococcaceae			14.86	14.60	1.12	0.87
		Rumminococcus		10.99	10.98	1.01	0.99
			*Ruminococcus gnavus*	5.55	3.29	0.59	< 0.01
			*R. obeum*	0.19	0.30	0.04	0.06
**Fusobacteria**				10.07	5.01	1.70	0.04
	Fusobacteriaceae			10.07	5.01	1.71	0.04
		Fusobacterium		0.74	0.48	0.11	0.09
			*Fusobacterium perfoetens*	0.47	0.19	0.12	0.10

1 Data are presented as percentage of sequences. CON  =  diets containing 0% cooked navy bean powder (n = 11); NB  =  diets containing 25% cooked navy bean powder (Vegeful ADM Edible Bean Specialties, Decatur, IL) (n = 10).

## Discussion

Dry beans have not been included in any commercial pet foods to date, thus if appropriate, it could be a novel protein, carbohydrate and fiber source. Forster et al. [7] reported no changes in serum chemistry measures, urinalysis, or digestibility in these dogs due to dietary treatment, indicating that cooked navy bean may be a viable petfood ingredient. This study evaluated the fecal microbiome of clinically healthy pet dogs before and after 4 wk of feeding macronutrient- and micronutrient-controlled diets containing either 0 or 25% dried navy bean powder. Few significant differences were observed in microbiome composition between 0 or 25% dried navy bean powder treatments after the 4 wk intervention; however, significant differences were observed due to dietary control (i.e., at baseline, prior to dietary control versus after 4 wk of dietary control). Regardless of diet, after 4 wk of treatment, the predominant bacterial phyla (Firmicutes, Actinobacteria, Fusobacteria) and genera within those phyla (Clostridium, Blautia, Collinsella, and Fusobacterium) were similar to those reported in the literature by other canine studies [1]. Given the similarities of the dietary treatments and the goals of this study, a large shift in microbial populations between CON and NB fed dogs was not expected, but minor changes were of interest if present.

*Blautia wexlerae*, a gram-positive bacteria, was increased by inclusion of cooked NB powder. Martinez [18] reported enrichment of this species in humans supplemented with 60 g of whole grain barley for 28-d compared to baseline (1.8 vs. 1.1%). Because *B. wexlerae* lacks enzymes with glucanase activity, it was hypothesized that whole grain barley inclusion impacted utilization of nutrients by bacteria at higher trophic levels resulting in increased substrate availability for *B. wexlerae*. Given these results, it may be of interest to examine the functional changes of the gastrointestinal microbiome. The authors are not aware of any research that links this bacterial species to negative or positive health consequences.

The proportions of *Clostridium saccharogumia* and *Coprobacillus cateniformis* were decreased in feces of dogs fed NB diets. The ability to metabolize lignans (i.e., polyphenolic glucosides) have been reported for *C. saccharogumia* and *Coprobacillus* species with > 90% sequence similarity to *C. cateniformis* [19], [20], [21]. For *C. saccharogumia*, this includes the capacity to metabolize the dietary phytoestrogen secoisolariciresinol diglucoside, which is high in wheat [19]. These data indicate that the decrease in these microbial species may be due to the decrease in dietary lignans from the wheat and corn [19]. The authors are not aware of any research that links this bacterial species to negative or positive health consequences.

The high variability at baseline and low number of animals may have masked small shifts in microbial populations due to navy bean inclusion. The micro- and macronutrient matching of the diets did allow us to determine that cooked navy bean inclusion elicited few alterations in fecal microbial populations. These data provide additional support for the safety of including dry beans as a viable staple pet food ingredient for canines. There are a number of studies supporting the chronic disease-fighting properties of dietary bean intake in humans and using rodent models [22], [23], [24], [25], [26]. The next step will be to assess bean diet-based changes during weight loss, in inflammatory conditions and with chronic diseases such as arthritis, diabetes, and cancer [8].

Micro- and macronutrient matching of the CON and NB diets allowed us to determine that navy bean as a source of carbohydrate, protein, fiber and phytochemicals did not alter the fecal microbial community. It is possible, however, that the diets may have been too similar to elicit large changes in microbial populations, and/or the small shifts which occurred were undetectable with this sample size, study design and technique utilized. For example, post-hoc estimation of the sample size necessary to detect differences due to diet for *Bifidobacterium*, *Lactobacillus, Clostridium, and Fecalibacterium* genus indicate that n = 51, 51, 55 and 82 dogs per treatment, respectively, would be needed [27]. Garcia-Mazcorro et al. [3] reported alterations in canine and feline fecal microbial populations after synbiotic administration with qPCR, DGGE, and gene clone library techniques; however, no change was noted when 454-pyrosequencing was utilized. These data indicate that more focused measurements may be needed to detect changes in fecal microbial populations when subtle changes in overall dietary nutrient or ingredient composition are assessed. Higher numbers of animals and possibly similar dog breeds per treatment groups, or large differences in dietary macronutrient composition may be necessary to detect alterations in the microbial populations as measured by 454-pyrosequencing in pet dogs.

Consistent with results from other studies, variability in the proportions of fecal microbial populations among dogs was high [3], [28]. The high variability likely limited our ability to discern differences due to 25% dietary navy bean intake. For example, the range of sequences that were identified as *Clostridium hiranonis* was 0.2 to 41% of sequences. Adding an initial adaptation period to studies in which all dogs are fed a similar diet may minimize variability in baseline measurements. Sample collection technique utilized herein (i.e., collected within 5 h of voiding) could have also introduced variability. Shorter windows of time from voiding to collection and subsequent freezing would increase consistency of sample handling and environmental exposure post voiding and potentially decrease variability. Another way to account for the variation in the study design in the future would be to provide diets in a cross-over design, so that each dog would act as its own control. Additionally, this would increase the number of dogs receiving each treatment. Many factors may potentially impact fecal bacterial populations (e.g., sex, breed, age, and diet) [1], [29]. Research utilizing pyrosequencing techniques to assess the fecal microbiome have primarily focused on either the impact of disease state or diet [1], thus, the relative importance of these factors is unknown. Eliminating these sources of variation (e.g., utilizing dogs from the same breed) could allow researchers to more easily detect the impacts of dietary treatments on microbial populations in pet dogs; however; utilizing animals with diverse life histories allows for the application of results to the general pet and even human population.

The alterations in fecal microbial populations due to dietary control may have obscured the ability to define changes due to dietary treatment. At baseline, owners reported feeding one or two of 14 different commercial diets, including 13 dry diets [guaranteed analysis (on a dry matter basis): 18.9 to 29.5% crude protein minimum, 7.8 to 17.8% crude fat minimum, 3.8 to 17.5% crude fiber maximum] and one wet diet [guaranteed analysis (on a dry matter basis): 39.9% crude protein minimum, 12.6% crude fat minimum, 2.2% crude fiber maximum]. Data are not available on the actual nutrient composition of these diets. Dietary protein, nitrogen free extract and fiber concentrations and sources, and the ratio of protein to carbohydrate (i.e., nitrogen free extract) have all been shown to alter microbial populations of cats and dogs [2], [4], [30], [31], [32], [33], For example, Middelbos et al. [4] reported data similar to ours for laboratory dogs fed diets containing 7.5% beet pulp (total dietary fiber: 4.5% DM) compared to control diets containing no supplemental fiber (total dietary fiber: 1.4% DM): The proportions of Actinobacteria (0.8 vs 1.4% of sequences) and Fusobacteria (24 vs 40% of sequences) were decreased (P<0.05) compared to control dogs, while Firmicutes were increased (28 vs 15% of sequences; P<0.05). There is growing evidence that the proportions of gastrointestinal microbes are altered in some disease states when compared with healthy dogs [28], [34], [35], [36]. The alteration of microbial populations herein, indicates that diet may have the potential to prevent dysbiosis in at risk dogs, or to shift microbial populations in diseased animals towards the profile of microbes in healthy animals. However, more research is needed to clearly define the profile of dysbiosis (i.e., how the microbial populations differ in diseased animal vs. healthy). Once these relationships have been defined, knowing the impacts of diets on microbial populations in healthy pet dogs will be invaluable. Additionally, these data may not only be useful for the pet population, but for human disease as well. There is emerging awareness and use of companion dogs as an advanced translational model for humans [37], [38].

## Conclusions

This study evaluated the fecal microbiome of healthy pet dogs before and after 4 wk of feeding macro- and micronutrient-controlled diets containing either 0 or 25% dried navy bean powder. Few significant differences were observed in microbiome composition between 0 and 25% cooked navy bean treatments after 4 wk. Continued research on the microbial metabolic impacts of dietary dry bean intake in dogs undergoing weight loss and with chronic diseases that are similar to human conditions is warranted.

Alterations in fecal microbial populations from baseline due to 4 wk dietary control macro- and micronutrient-controlled diets were noted. These data highlight the potential to alter microbial populations of pet dogs with diverse backgrounds and microbial populations in the same direction. The proportions of gastrointestinal microbes are altered in dogs with some diseases compared to healthy animals (e.g., acute hemorrhagic diarrhea, non-hemorrhagic diarrhea, and irritable bowel disease). Understanding the impacts of dietary changes on microbial populations in healthy dogs may help to treat or prevent these diseases in the future.

The proportions of microbes are highly variable in pet dogs, which may impact the ability to detect differences when treatments are similar. Studies performed in pet dogs utilizing 454-pyrosequencing should attempt to decrease variation, or pair 454-pyrosequencing with targeted assays (e.g., qPCR, gene clone libraries, etc.). Variation would likely have been decreased in this study by utilizing a cross-over design, adapting animals to the control diet prior to baseline measurements, or selecting animals with more similar life histories (i.e., sex, breed, age, and previous diet).
